# A hypothesis-free approach to identifying potential effects of relative age in school year: an instrumental variable phenome-wide association study in the UK Biobank

**DOI:** 10.1093/aje/kwae331

**Published:** 2024-08-31

**Authors:** Melanie A de Lange, Neil M Davies, Louise A C Millard, Kate Tilling

**Affiliations:** MRC Integrative Epidemiology Unit, Bristol Medical School, University of Bristol, Bristol, United Kingdom; Population Health Sciences, Bristol Medical School, University of Bristol, Bristol, United Kingdom; Division of Psychiatry, University College London, London, United Kingdom; Department of Statistical Sciences, University College London, London, United Kingdom; K.G. Jebsen Center for Genetic Epidemiology, Department of Public Health and Nursing, Norwegian University of Science and Technology, Trondheim, Norway; MRC Integrative Epidemiology Unit, Bristol Medical School, University of Bristol, Bristol, United Kingdom; Population Health Sciences, Bristol Medical School, University of Bristol, Bristol, United Kingdom; MRC Integrative Epidemiology Unit, Bristol Medical School, University of Bristol, Bristol, United Kingdom; Population Health Sciences, Bristol Medical School, University of Bristol, Bristol, United Kingdom

**Keywords:** relative age, hypothesis-free, IV-pheWAS, summer-born, birth date

## Abstract

A child’s relative age within their school year (“relative age”) is associated with educational attainment and mental health. However, hypothesis-driven studies often re-examine the same outcomes and exposure, potentially leading to confirmation and reporting biases and missing unknown effects. Hypothesis-free outcome-wide analyses can potentially overcome these limitations. We conducted a hypothesis-free investigation of the effects of relative age within school year. We performed an instrumental variable (IV) phenome-wide association study in the UK Biobank (participants aged 40-69 years at baseline), using the PHESANT software package. We created 2 IVs for relative age: being born in September vs August (*n* = 64 075) and week of birth (*n* = 383 309). Outcomes passing the Bonferroni-corrected *P* value threshold for either instrument were plotted to identify a discontinuity at the school year transition. Thirteen traits associated with at least 1 of the instruments showed a discontinuity. Previously identified effects included those with a younger relative age being less likely to have educational qualifications and more likely to have started smoking at a younger age. We detected a few associations not explored by previous studies. For example, those of younger relative age had better lung function as adults. Hypothesis-free approaches could help address confirmation and reporting biases in epidemiology.

## Introduction

Children’s age within their school year (henceforth relative age) consistently relates to educational attainment. In the United Kingdom, children are rarely held back a school year, so age relative to September 1 is almost synonymous with age within school year. Children born later in the school year have lower average educational attainment than those born at the beginning of the school year.^1^^-^^6^ In England, where entry for each school year runs from September 1 to August 31, summer-born children, on average, perform worse than autumn-born children.^7^

Relative age differences in educational attainment occur across a range of education systems with different school year start dates and so are unlikely to be due to season of birth.^8^ Although educational differences are greatest when comparing those born at the start with those born at the end of the school year (eg, September vs August in England), there is evidence of a linear change in educational outcomes over relative age.^7^ These differences are largest when children start school and diminish with age, but they are still evident when children finish compulsory schooling.^7^ Children born later in the school year are also more likely to be diagnosed with special educational needs,^9^^-^^14^ be bullied at school,^10^^,^^15^ engage in risky behavior such as underage smoking,^7^ and commit a crime in adolescence.^16^ They also tend to have poorer mental health during childhood^14^^,^^17^^-^^19^ and higher suicide rates in adolescence and young adulthood (ages 15-25 years).^20^ A limited number of studies have examined whether the effects of relative age persist into adulthood. Some studies have found negative effects relative to adults’ employment status,^21^ their earnings,^22^^-^^24^ self-confidence, and risk-taking.^25^ Others have found little effect on whether people are employed and their earnings,^7^^,^^21^^,^^26^^,^^27^ or health and mental well-being during adulthood.^7^^,^^19^^,^^21^

To date, research investigating relative-age effects has focused on a few outcomes, mostly educational or related to mental health and well-being. In contrast to such hypothesis-driven approaches that test the association of a specific exposure with a specific outcome (or a small targeted number of each), hypothesis-free approaches test all measured factors, regardless of previous knowledge, to search for associations even where there is no prior suggestion that an association may exist.^28^ In general, hypothesis-free approaches are exploratory approaches: the intention is to identify potential associations and then follow up (including with replication) in a hypothesis-driven manner.

Phenome-wide association studies (PheWAS) are a type of hypothesis-free analysis that can identify novel associations by testing the association of a trait of interest with many other phenotypes (“the phenome”).^29^ PHESANT is a software package for performing comprehensive phenome scans in UK Biobank data.^29^ Rather than being limited to a homogenous subset of phenotypes that can be analyzed in the same way, PHESANT enables users to scan a heterogenous set of phenotypes (all UK Biobank continuous, integer, and categorical fields).^29^

Instrumental variable (IV) analysis is a technique used to deal with unmeasured confounding in observational studies. An IV is a variable related to the exposure that is only related to the outcome via the exposure, and can be used to infer causality.^30^ Although IVs are often used in a 2-stage modeling approach to estimate the magnitude of effect of an exposure of interest on the outcome, the effect of the IV on the outcome can also be estimated and is a valid test for the presence of an effect of the exposure on the outcome.^31^ To date, PHESANT has largely been used to conduct Mendelian randomization pheWAS, where the IV is a genetic variant or genetic risk score.^32^ However, it can also be used with other types of IVs. In general, we use the term IV-pheWAS to refer to a hypothesis-free scan that uses an IV to search for the causal effect of an exposure across many outcomes. Furthermore, although we use an IV framework, we note that this analysis could also be formulated as a regression discontinuity design, and many previous studies of relative school age have taken this approach (see Appendix S1 for further details).^19^^,^^20^

In this study, we used PHESANT to conduct 2 IV-pheWAS to identify phenotypes associated with relative age among approximately 383 000 UK Biobank participants born in England. We aimed to identify both the short-term effects of relative age while in school, as well as more long-term impacts that persist into later life.

## Method

### Study population

The UK Biobank is a prospective cohort of approximately 500 000 adults who were aged mostly 40-69 years when recruited in 2006-2010^33^ (5.5% participation rate^34^). The UK Biobank contains extensive phenotypic data collected via lifestyle and health questionnaires. These cover the period from birth to recruitment and include sociodemographics, family history and early life exposures, environmental factors (eg, occupation, housing), lifestyle factors (eg, exercise, smoking, alcohol), cognitive function, and medical history. The UK Biobank also contains data on physical measures such as blood pressure, grip strength, bone density, and eye measurements, as well as blood, saliva, and urine samples.^33^ This research was conducted under UK Biobank application 16729 (data set identifier 48196).

**Figure 1:**
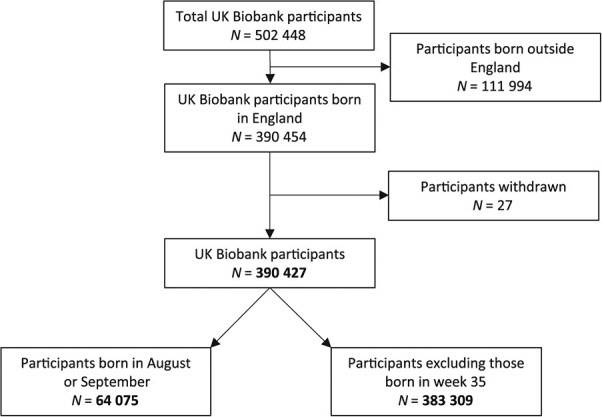
Participant flow diagram.

Of the 502 448 UK Biobank participants, we removed 111 994 participants who were not born in England (other UK countries have different school entry cutoff dates). We also removed 27 participants who withdrew their consent, leaving a final sample of 390 427 (Figure 1).

### IVs for relative age

Month and week of birth information was acquired from a central registry and updated by participants at their initial UK Biobank assessment center visit. These variables were used to create 2 IVs for relative age. The IV for birth in September vs August (IV_Sep-Aug_) was a binary variable restricted to participants born in September or August, with September coded as 1 and August as 0. The IV for week of birth (IV_weeks_) was an integer variable, with the first full week in September (calendar week 36) coded as 1 and the last full week of August (calendar week 34) coded as 51. We excluded calendar week 35 (containing the August-September crossover) to avoid misclassification (because we cannot know if these participants were born in August or September). Although we have called our exposure relative age within school year, relative age is, in fact, a compound treatment comprising multiple treatments, including relative age in the classroom, time spent in preschool, and years in school before compulsory school age. It is not possible to disentangle these treatments. Therefore, although we refer to our exposure as relative age in school year, our approach should be seen as an intention-to-treat estimate of the entire compound treatment.

Instrumental variables are defined by 3 assumptions: (1) they are associated with the exposure (relevance assumption), (2) they have no common cause with the outcome (independence assumption), and (3) they only affect the outcome through the exposure (exclusion restriction assumption).^31^^,^^35^  Figure S1 is a directed acyclic graph of the assumptions underpinning our IV analysis. In England, children are assigned to a school year on the basis of their date of birth, with a cutoff on September 1. Therefore, a child’s relative school age is determined by their date of birth, which will be associated (indeed be virtually synonymous) with relative school age. This differs from other education systems, such as in the United States, where it is more common for children to be held back a year, and thus for relative school age to differ even for children who have the same date of birth. In this study, the exposure (relative age in school year) is not measured—for each participant, we do not know how old they were when they started school. However, IVs can provide a valid test of the null hypothesis of no effect of the exposure even if the exposure is unmeasured.^31^ To deduce the direction of the effect of the exposure on the outcome, we need to make 1 more assumption: we assume monotonicity of the IV to exposure relationship; that is, there are no people who would have started school a calendar year later if they were born in August than if they had been born in September of the same year.

To investigate whether our instruments were associated with potential confounders of the relationship between relative age and our outcomes, we ran regression analyses of birth year, sex, birth weight, region, and our IV_Sep-Aug_ variable. We tested whether the transition between school years (August/September) acted via seasonality rather than the exposure (relative school age) (ie, whether assumption 3, the exclusion restriction assumption, was likely to be satisfied). Estimates and CIs for the IV for month of birth relative to September were plotted for each potential effect and these were manually checked by the authors to identify whether a discontinuity at the August vs September cutoff was apparent.

### Covariates

We included age at recruitment and sex as covariates in our models to reduce variation in our outcomes and increase precision. Age was derived from date of birth and date of attending initial assessment, and provided to us by UK Biobank as an integer. Sex was acquired from the National Health Service central registry at recruitment but was updated by the participant in some cases.

### Deriving outcomes with PHESANT

The UK Biobank Showcase (https://biobank.ndph.ox.ac.uk/showcase/) provides a searchable directory of variables available in the UK Biobank based on field type (integer, continuous, categorical [single], and categorical [multiple]). PHESANT was run with the “save” option on to derive cleaned variables to be used as outcomes in the IV-pheWAS. This involved PHESANT categorizing each outcome variable into 1 of 4 data types: continuous, ordered categorical, unordered categorical, or binary, using its automated rule-based method described elsewhere.^29^ The derived outcomes were stored as CSV files labeled according to data type.

### Statistical methods

#### IV-pheWAS

The analysis plan was developed by 2 of our team (L.A.C.M. and K.T.) prior to starting our analyses. We performed an IV-pheWAS for both IV_Sep-Aug_ and IV_weeks_. Each IV-pheWAS tested the association of the IV directly with each derived outcome variable. A total of 15 034 outcomes were tested for IV_Sep-Aug_ and 23 164 outcomes were tested for IV_weeks_. Regression analyses were adjusted for age and sex. Ordered logistic regression was used to test associations with ordered categorical outcome variables, multinomial logistic regression for unordered categorical outcome variables, binomial logistic regression for binary outcome variables, and linear regression for continuous outcome variables. For continuous outcomes, estimates of effect size produced are the mean difference of inverse rank normal transformed outcomes for September vs August-born, and can be interpreted as a 1-unit change in the inverse rank normal transformed variables. For binary outcomes, the estimates are log odds of outcomes for September vs August-born and for categorical ordered outcomes the estimates are log odds of being in a higher outcome category for September vs August-borns.

We only tested outcomes with at least 500 participants and with at least 10 participants in each category in binary and unordered categorical variables, because phenotypes that did not meet these thresholds were unlikely to be sufficiently powered. *P* values were generated for each outcome, using a likelihood ratio test comparing the model including the IV as a covariable vs the same model but not including the IV as a covariable. Results were ranked according to *P* value. To take multiple testing into account when evaluating the strength of the evidence of our results, we defined potential effects as any association of relative age with an outcome that passed the Bonferroni-corrected significance threshold (0.05 divided by the number of tests performed) in either IV-pheWAS. Doing so controls the family-wise error rate by making a conservative assumption that tests are independent.^36^

We also ran a third IV-pheWAS, the IV for month of birth relative to September, where our IV was a set of 11 indicator variables describing the month of birth of each participant (excluding September) and compared each of the 11 months with born in September. However, we have not included the third IV-pheWAS in our main results, because it picked up many associations not specific to the discontinuity we were looking for (see Table S1 for results).

Analyses were performed in R, version 4.1.0, and Stata, version 17. Code is available at https://github.com/MRCIEU/PHESANT-IV-pheWAS-relative-school-age (git tag v0.2 corresponds to the version presented).

## Results

### Test of IV assumptions

Logistic regressions to test whether our IV_Sep-Aug_ variable was associated with potential confounders of the relationship between relative age and our outcomes showed that sex, birth weight, and UK Biobank assessment center were not associated with relative age (Table S2). Year of birth was very weakly negatively associated with relative age (for being born in September vs August per 1-year increase in birth year, odds ratio [OR] = 0.9979; 95% CI, 0.9960-0.9998). However, we adjusted for age at recruitment to UK Biobank (whole years) in our analyses.

### IV-pheWAS results

Of the 15 034 tests performed for IV_Sep-Aug_ and 23 164 tests performed for IV_weeks_, we found 21 traits that had a *P* value below the Bonferroni threshold in at least 1 of these IV-pheWAS (Table 1; Table S1 presents full results for both IVs [note we do not have estimates for unordered categorical variables]). The 8 traits associated with IV_Sep-Aug_ were also associated with IV_weeks_. Model convergence was not reached for a minority of outcomes; as a result, these outcomes were not considered further (see Table S3 for a list of these variables).

**Table 1:** Traits associated with being born in September vs August or week of birth.

**Outcome**	**Outcome type**	**Total no.** ^a^	***P* value for IV**_**Sep-Aug**_	***β* (95% CI) for IV**_**Sep-Aug**_	**Total no.** ^a^	***P* value for IV**_**weeks**_	***β* (95% CI) for IV**_**weeks**_	**Discontinuity at school year transition evident on forest plot**
Age completed full-time education^b^	Ordered categorical	43 933	7.24 × 10^−162^	.48 (.45-.52)	261 871	4.81 × 10^−173^	−.007 (−.008 to −.007)	✓
Comparative height at age 10 years^c^	Ordered categorical	63 039	1.25 × 10^−141^	.39 (.36-.42)	376 987	8.55 × 10^−295^	−.008 (−.008 to −.007)	✓
Year ended full-time education	Continuous	16 008	1.96 × 10^−30^	.08 (.06-.09)	96 107	1.03 × 10^−41^	−.001 (−.001 to −.001)	✓
Qualifications: CSEs or equivalent (vs not have CSEs or equivalent)	Binary	62 941 (53 530 vs 9411)	1.22 × 10^−25^	−.25 (−.30 to −.20)	376 822 (320 620 vs 56 202)	4.71 × 10^−60^	.005 (.005-.006)	✓
Qualifications: O levels/GCSEs or equivalent (vs not having O levels/GCSEs)	Binary	62 941 (32 791 vs 30 150)	1.84 × 10^−14^	.12 (.09-.16)	376 822 (195 116 vs 181 706)	1.01 × 10^−17^	−.002 (−.002 to −.002)	✓
Qualifications: college or university degree (vs not having college or university degree	Binary	62 941 (43 794 vs 19 147)	4.06 × 10^−9^	.10 (.07-.14)	376 822 (261 214 vs 115 608)	3.03 × 10^−7^	−.001 (−.002 to −.001)	✓
Age started smoking in former smokers	Continuous	15 647	6.03 × 10^−9^	.09 (.06-.12)	94 093	3.86 × 10^−7^	−.001 (−.002 to −.001)	✓
Qualifications: A/AS levels or equivalent (vs not having A/AS levels or equivalent)	Binary	62 941 (46 190 vs 16 751)	4.72 × 10^−8^	.10 (.06-.14)	376 822 (275 200 vs 101 622)	3.93 × 10^−7^	−.001 (−.002 to −.001	✓
Sensitivity/hurt feelings: Are your feelings easily hurt? (yes or no)	Binary	62 277 (27 659/34618)	.27	−.02 (−.05 to .01)	372 349 (164 547 vs 207 802)	2.54 × 10^−25^	.002 (.002-.003)	
Year job started (calendar year)	Continuous	15 892	4.51 × 10^−6^	.05 (.03-.07)	95 445	2.66 × 10^−10^	−.001 (−.001 to −.001)	✓
Ever had bowel cancer screening (vs never having had bowel cancer screening)	Binary	63 077 (42 549/20528)	.75	−.01 (−.04 to .03)	377 262 (253 638 vs 123 624)	4.93 × 10^−9^	.002 (.001-.002)	
3 mm asymmetry angle (right)	Continuous	14 306	.02	.04 (.01-.07)	85 613	4.44 × 10^−8^	−.001 (−.002 to −.001)	
Mean MO in tapetum on FA skeleton (right), from brain MRI	Continuous	5595	.06	−.05 (−.10 to .003)	33 361	1.09 × 10^−7^	.002 (.001-.003)	
Mean OD in tapetum on FA skeleton (right), from brain MRI	Continuous	5595	.06	.05 (−.002 to .10)	33 359	2.75 × 10^−7^	−.002 (−.003 to −.001)	
Trouble concentrating in last 2 weeks^d^	Ordered categorical	21 750	.01	−.09 (−.15 to −.02)	131 316	3.83 × 10^−7^	.002 (.001-.003)	✓
Mood swings: does your mood often go up and down? (yes vs no)	Binary	62 557 (34 669/27888)	.05	−.03 (−.06 to .0004)	374 169 (204 225 vs 169 944)	3.88 × 10^−7^	.001 (.001-.002)	
Forced expiratory volume in 1 s, *z* score (spirometry)	Continuous	50 776	.02	−.02 (−.04 to −.004)	303 772	4.19 × 10^−7^	.001 (.0003-.001)	✓
Mean MO in inferior cerebellar peduncle on FA skeleton (left)	Continuous	5595	.07	.05 (−.003 to .10)	33 361	4.43 × 10^−7^	−.002 (−.003 to −.001)	
Neuroticism score^e^	Ordered categorical	52 115	.64	.007 (−.02 to .04)	310 355	1.17 × 10^−6^	.001 (.001-.001)	
Job code: typist (vs not having a job code of typist)	Binary	15 684 (15 141/543)	.002	−.27 (−.45 to −.10)	94 121 (90 882 vs 3239)	1.58 × 10^−6^	.006 (.004-.009)	✓
Historical job code: typist (vs not having a historical job code of typist)	Binary	15 684 (15 141/543)	.002	−.27 (−.45 to −.10)	94 121 (90 882 vs 3239)	1.58 × 10^−6^	.006 (.004-.009)	✓

Abbreviations: CSE, Certificate of Secondary Education; FA, fractional anisotropy; GCSE, General Certificate of Secondary Education; IV_Sep-Aug_, September vs August birth date as an instrumental variable; IV_weeks_, week of birth as an instrumental variable; MO, diffusion tensor mode; MRI, magnetic resonance imaging; OD, orientation dispersion.Continuous outcome: estimates are mean difference of inverse rank normal transformed outcome for September versus August born, and can be interpreted as a 1-unit change in the inverse rank normal transformed variables. Binary outcome: estimates are log odds of outcome September versus August born. Categorical ordered outcome: estimates are log odds of being in a higher outcome category for September versus August born. Estimates in grey are those that did not pass the Bonferroni correction for that IV-pheWAS.

^a^The number in each category for binary outcomes.

^b^Age categories: <16 years, 16 years, ≥17 years.

^c^Height categories: shorter, about average, taller.

^d^Categories: not at all, several days, more than half the days, nearly every day.

^e^12 categories: 0 to 12.

Six of the 8 traits that passed the Bonferroni-corrected threshold (*P* = 3.33 ×10^−6^) for IV_Sep-Aug_ were related to education (Table 1). For example, participants born in September were more likely to have O levels or a General Certificate of Secondary Education (GCSE) (*P* = 1.84 ×10^−14^; OR = 1.13; 95% CI, 1.10-1.17), advanced/advanced subsidiary (A/AS) levels (*P* = 4.72 ×10^−8^; OR = 1.10; 95% CI, 1.07-1.15), or a college/university degree (*P* = 4.06 ×10^−9^; OR = 1.11; 95% CI, 1.07-1.15), and less likely to have a Certificate of Secondary Education (CSE) (*P* = 1.22 ×10^−25^; OR = 0.78; 95% CI, 0.74-0.82) than those born in August. Those born in September were also more likely to complete full-time education at an older age than those born in August (*P* = 7.24 ×10^−162^; OR = 1.62; 95% CI, 1.57-1.68). These findings are supported by the results of IV_weeks_, which indicated participants born in a later week in the academic year had worse education outcomes (Table 1).

Moving beyond education, participants born in September vs August and participants with an earlier week of birth in school year were more likely to report being comparatively taller for their age at age 10 years (for IV_Sep-Aug_: *P* = 1.25 ×10^−141^, OR = 1.47 [95% CI, 1.43-1.52]; for IV_weeks_: *P* = 8.55 ×10^−295^, OR = 0.992 [95% CI, 0.992-0.993]). Participants born in September vs August who are former smokers also reported starting smoking later (*P* = 6.03 ×10^−9^; β = .09; 95% CI, .06-.12). Those with a later week of birth in school year (younger relative age) also started smoking at an earlier age (IV_weeks_: *P* = 3.86 ×10^−7^; β = −.001; 95% CI, −.002 to −.001). However, little evidence of an association between relative age and the age at which they started smoking was seen among current smokers (for IV_Sep-Aug_: *P* = .32, β = .03 [95% CI, −.03 to .08]; for IV_weeks_: *P* = .16; β = −.0005 [95% CI, −.0013 to .0002]).

Another 13 traits passed the Bonferroni-corrected threshold (*P* = 2.16 ×10^−6^) for IV_weeks_ alone. In terms of psychological traits, IV_weeks_ was positively associated with having one’s feelings hurt easily (*P* = 2.54 ×10^−25^; OR = 1.002; 95% CI, 1.002-1.003), having mood swings (*P* = 3.88 ×10^−7^; OR = 1.0012; 95% CI, 1.0007-1.0016), and participants’ neuroticism scores (*P* = 1.17 ×10^−6^; OR = 1.0010; 95% CI, 1.0006-1.0015), meaning those with a younger relative age (ie, later week of birth) were more likely to have these traits. Week of birth also was associated with some physical measurements, including being positively associated with forced expiratory volume in 1 second *z* score (*P* = 4.19 ×10^−7^; β = .0006; 95% CI, .0004-.0009). There also was a negative association for IV_weeks_ with 3-mm asymmetry angle (right eye; a measure of astigmatism) (*P* = 4.44 ×10^−8^; β = −.0013; 95% CI, −.0017 to −.0008), mean orientation dispersion in the tapetum on right-side fractional anisotropy skeleton (*P* = 2.75 ×10^−7^; β = −.0019; 95% CI, −.0026 to −.0012), and mean diffusion tensor mode in the inferior cerebellar peduncle on the left-side fractional anisotropy skeleton (*P* = 4.43 ×10^−7^; β −.0018; 95% CI, −.0025 to −.0011), and a positive association with mean diffusion tensor mode in tapetum on the right-side fractional anisotropy skeleton (1.09 ×10^−7^; β = .0019; 95% CI, .0012-.0027).

There was a positive association for IV_weeks_ with ever being screened for bowel cancer (*P* = 4.93 ×10^−9^; OR = 1.0015; 95% CI, 1.0010-1.0020) but little evidence of an association with people reporting having had bowel cancer itself (for small bowel cancer: *P* = .89, OR = 1.0008 [95% CI, 0.9905-1.0111]; for large bowel cancer: *P* = .19, OR = 0.9961 [95% CI, 0.9903-1.0020]). There was a positive association for IV_weeks_ with having a job code or historical job code of typist (*P* = 1.58 ×10^−6^; OR = 1.0061; 95% CI, 1.0036-1.0086 for both). We found IV_weeks_ was also positively associated with answering yes to the question “Over the last 2 weeks, how often have you been bothered by trouble concentrating on things, such as reading the newspaper or watching television? [experience of pain]” (*P* = 3.83 ×10^−7^; OR = 1.002; 95% CI, 1.001-1.003). Another related phenotype, “Over the last 2 weeks, how often have you been bothered by any of the following problems? [depressive symptoms] Trouble concentrating on things, such as reading the newspaper or watching television,” was associated in a direction consistent with our finding, although it did not pass the Bonferroni threshold (*P* = .03; OR = 1.0011; 95% CI, 1.0001-1.0021).

### Meta-analysis forest plots

Of the 21 potential effects identified in the IV-pheWAS, 20 had an output from PHESANT for the IV for month of birth relative to September and were plotted by month of birth. A discontinuity at the transition point between school years was visually identified for 13 of the 20 (Table 1, Figures S2-21): educational qualifications (CSEs, O level/GCSEs, A/AS levels, college/university degree), the year and age when full-time education ended, the year a person’s job started, comparative height at age 10 years, the age former smokers started smoking, having trouble concentrating in the past 2 weeks, forced expiratory volume in 1 second *z* score, and having a job code or historical job code of a typist.

## Discussion

In this study, we conducted hypothesis-free IV-pheWAS to explore the effects of relative age in school year on approximately 383 000 UK Biobank participants born in England. We found 21 phenotypes associated with relative age, 13 of which displayed a discontinuity at the transition between school years. We identified both the short-term effects of relative age while in school, as well as the more long-term impacts persisting into later life that relate to children being penalized for appearing to be less skilled than their peers in school.

In terms of the effects of relative age during school, we found that younger relative age is associated with a lower likelihood of having educational qualifications, with 10%-13% higher odds of those born in September having GCSEs/A levels or a degree than those born in August. This is consistent with previous studies that report an association between younger relative age and lower educational attainment.^1^^-^^7^

We also identified other logical effects of relative age while children are in school. These include having an older relative age being associated with reporting being taller than peers (where participants likely think of their school year as their peers), with 47% higher odds of those born in September reporting being taller than their peers at age 10 years than those born in August. Furthermore, those with a younger relative age are marginally more likely to start smoking at an earlier age (among former smokers), with the September-born participants being 0.09 years older than those born in August when they started smoking. This is also somewhat expected given that peers are likely to start smoking at the same time, so those younger in the year will be younger when they start.

Looking at the persistence of relative age effects into adulthood, in contrast to some previous studies^22^^-^^24^, but in line with others,^7^^,^^21^^,^^26^^,^^27^ we found little evidence of long-term effects on employment prospects or earnings; our only potential effect was week of birth being associated with having a job code or historical job code of a typist. However, the effect sizes were too small to be meaningful (odds of being a typist per 1-week increase in date of birth was 1.0061 times higher). In addition, we did not find evidence of an effect of relative age on current employment status, but this may be because approximately one-third of UK Biobank participants are retired, so this variable is not necessarily a measure of occupational success. Nevertheless, a higher average total household income was also associated with being born in September vs August and week of birth (for IV_Sep-Aug_: *P* = 1.66 ×10^−4^, OR = 1.06 [95% CI, 1.03-1.09]; for IV_weeks_: *P* = 7.73 ×10^−4^, OR = 0.9993 [95% CI, 0.9989-0.9997]), although it did not pass the Bonferroni threshold of either IV-pheWAS. Furthermore, in accordance with existing research,^7^^,^^19^^,^^21^ we also found little evidence of long-term effects on adult mental health, with mental health phenotypes that passed the Bonferroni threshold, such as sensitivity and mood swings, not displaying a discontinuity when plotted.

Previous research into the effect of relative age on adult physical health found no substantial differences in self-reported health.^19^ In line with this, our IV-pheWAS only found 1 potential effect related to physical health. We found that adults with a younger relative age performed better on a forced expiratory lung function test. However, the effect size was too small to be clinically meaningful (a 1-week increase in date of birth was associated with a 0.0006 increase in forced expiratory volume in 1 second z score). Nevertheless, this result is supported by the fact that when we searched our results, we also found that forced vital capacity *z* score (another measure of lung function) was also positively associated with week of birth (*P* = 5.97 ×10^−5^; β = .0005; 95 CI, .0003-.0007), meaning those with a younger relative age had a greater capacity. However, this did not pass the Bonferroni threshold and the effect size was too small to be meaningful. The effect of relative age on adult lung function requires further exploration in other data sets to confirm whether the effect observed here is a true effect.

Our other potential effect of relative age on adult outcomes was that those with a younger relative age were more likely to have had trouble concentrating in the past 2 weeks (experience of pain). However, the effect size was too small to be meaningful, with the odds of having problems concentrating per 1-week increase in date of birth being 1.002 times higher. Nonetheless, we also found having had “recent trouble concentrating on things” (depressive symptoms) was associated with relative age (albeit beyond the Bonferroni threshold), with the odds of having recent problems concentrating per 1-week increase in date of birth being 1.0011 times higher. Given that relative age previously has been associated with childhood diagnosis of attention-deficit/hyperactivity disorder,^11^^-^^14^ the effects of relative age on concentration in adulthood could warrant further investigation.

A key strength of our hypothesis-free IV-PheWAS approach is that it enables potentially novel effects to be uncovered, thus revealing new research avenues. This possibility is amplified by the fact that the UK Biobank includes an extensive number and range of phenotypes and that the PHESANT software allowed us to perform comprehensive phenome scans, rather than our set of outcomes being restricted to a homogenous subset.^29^ We used the Bonferroni-corrected *P* value threshold to keep the study scope manageable and reduce the likelihood of identifying effects that are due to chance. However, this method may be overly strict and, therefore, could have potentially resulted in our analyses missing some outcomes where relative age does have a true small effect. Full results using the false discovery rate threshold are provided for follow-up in future studies (Table S1).

Although the hypothesis-free nature of our study has advantages, the indiscriminate nature of our IV-pheWAS gave a high multiple testing burden and so likely missed several true effects.

 Further limitations of this work include that the UK Biobank is not representative of the general UK population, with participants tending to be older, healthier, female, and from less socioeconomically deprived areas.^34^ If relative age in school year also affects participation in UK Biobank (which is likely, given its suggested effect on educational attainment, which, in turn, is strongly associated with UK Biobank participation), then this could cause selection bias in estimating causal effects of relative age.

Instrumental variable analyses require 3 core IV assumptions to be met. The relevance assumption was not testable here. However, given the close correspondence between our IV definitions and date of birth, it seems reasonable to assume these are strongly associated with relative age in the school year. We tested the independence assumption by examining the association of IV_Sep-Aug_ with potential confounders. We also tested the exclusion restriction assumption, but could not do so for pathways other than seasonality. As already described, our study looks at the intention-to-treat effect of relative age and should be seen as an estimate of the whole compound treatment. It was not possible to disentangle effects of the individual components (eg, relative age in the classroom vs time spent in preschool). For further discussion of our testing of IV assumptions, see Appendix S2.

PHESANT uses an automatic rule-based method to decide how each outcome is tested, and it is possible that this may treat some variables inappropriately. In addition, models did not converge for a small number of traits, so their relationship with relative age could not be reported. Last, because pheWAS aim to generate hypotheses, associations discovered may have been a chance finding or due to violations of the IV assumptions. Therefore, it is important that future work replicates the results in an independent sample and triangulates them using other study designs that have contrasting assumptions and biases.^37^ Furthermore, results may not be generalizable to other populations, such as education systems outside of the United Kingdom; hence, further studies in other populations are needed to explore this.

In summary, this study adds to the body of evidence that suggests children born later in the academic year have lower educational attainment than those born earlier in the school year. However, using a hypothesis-free approach, we detected associations not explored by previous studies. Our findings require replication, and the policy and clinical implications of our research should be assessed in the context of the overall literature.^38^ Future research should examine our identified effect on lung function in different populations and using different study designs, and investigate the mechanisms through which this effect may act. The medium-term consequences of relative age on career prospects and physical and mental health in early adulthood could also be investigated. Hypothesis-free approaches are a valuable exploratory method that complements more traditional hypothesis-driven studies and could help overcome confirmation and reporting biases in epidemiology.

## Acknowledgments

This manuscript was previously published as a preprint in medRxiv (https://doi.org/10.1101/2023.05.26.23290586). This research was conducted using the UK Biobank Resource under application no. 16729. We thank Dr. Beate Leppert for contributing to the early stages of this work. This work was carried out using the computational facilities of the Advanced Computing Research Centre, University of Bristol (http://www.bristol.ac.uk/acrc/). For the purpose of open access, the authors have applied a CC BY public copyright license to any Author Accepted Manuscript version arising from this submission. Author Contributions: Louise A. C. Millard and Kate Tilling contributed equally to this work.

## Supplementary Material

Web_Material_kwae331

## Data Availability

The UK Biobank data set used to conduct the research reported here is available via application directly to the UK Biobank. Applications are assessed for meeting the required criteria for access, including legal and ethics standards. More information regarding data access can be found at https://www.ukbiobank.ac.uk/enable-your-research. Analyses were performed in R, version 4.1.0, and Stata, version 17. Code is available at https://github.com/MRCIEU/PHESANT-IV-pheWAS-relative-school-age (git tag v0.2 corresponds to the version presented).
