# Supplementary material for: A hypothesis-free approach to identifying potential effects of relative age in school year: an instrumental variable phenome-wide association study in the UK Biobank
Source: Am J Epidemiol. 2024 Aug 31;194(6):1673–80. doi: 10.1093/aje/kwae331 (PMC12133288; doi:10.1093/aje/kwae331)
Supplement: Web_Material_kwae331 [file web_material_kwae331.zip › Supplementary Material.docx]

**Supplementary Material**

**A hypothesis-free approach to identifying potential effects of relative age in school year: an instrumental variable phenome-wide association study in the UK Biobank**

Melanie A de Lange, Neil M Davies, Louise AC Millard, Kate Tilling

**Table of Contents:**

Appendix S1: Regression Discontinuity

Appendix S2: Testing IV Assumptions

Figure S1: Directed Acyclic Graph of the Assumptions Underpining our Instrumental Variable Analysis

Figures S2-S21: Forestplots for all outcomes that passed the Bonferroni threshold for IVsep-aug or IVweeks by month of birth vs September (IVmonths)

Table S2: Logistic Regression Results to Compare the Covariates of People Born in August vs September.

Appendix S1: Regression Discontinuity Design

Many studies of relative age have used a regression discontinuity design (RDD). This is a quasi-experimental method that can be used be used to examine the effects of policies which assign people to a treatment or intervention based on a threshold on a continuous variable^1, 2^. Because a child’s birth date cannot be controlled precisely (assuming natural birth), whether a child is assigned to the group born before or after the school year entry cut-off is quasi-random^3^. This means that, close to the threshold, covariates should be balanced between those born just before or just after the cut-off^3^. Thus, the RDD approach can give unbiased estimates of causal effects if the window around the threshold is small, and the underlying continuous variable (here the date of birth) cannot be manipulated^1^. Although this is often referred to in the literature as a RDD design (with 1st September as the cut-off), this idiosyncratic event can also be analysed using the IV framework with date of birth (compared to the September cut-off) as the IV.

References

1. Moscoe E, Bor J, Bärnighausen T. Regression discontinuity designs are underutilized in medicine, epidemiology, and public health: a review of current and best practice. *J Clin Epidemiol*. 2015;68(2):132-43. <https://doi.org/10.1016/j.jclinepi.2014.06.021>.

2. Bor J, Moscoe E, Mutevedzi P, Newell M-L, Bärnighausen T. Regression discontinuity designs in epidemiology. *Epidemiology*. 2014;25(5):729-37. <https://doi.org/10.1097/ede.0000000000000138>.

3. Broughton T, Langley K, Tilling K, Collishaw S. Relative age in the school year and risk of mental health problems in childhood, adolescence and young adulthood. *J Child Psychol Psychiatry*. 2023;64(1):185-96. <https://doi.org/10.1111/jcpp.13684>.

Appendix S2: Testing of IV Assumptions

To test for an effect of relative age, we have used IV analyses that assume that the three core IV assumptions hold. At least one of the assumptions may be violated, and whether the independence and exclusion restriction assumptions are violated may vary across outcome variables. The relevance assumption, that the IV is strongly associated with the exposure, is not testable in our sample as we do not have access to the participant’s date of starting school, only the week, month, and year of their birth. However, given the close correspondence between our IV definitions and the date of birth, it is reasonable to assume these are strongly associated with relative age in the school year. To assess whether the independence assumption holds, we tested the association of the IVsep-aug variable with potential confounders: year of birth, sex, and UK Biobank assessment centre. These were selected as they could not reasonably be affected by relative age in the school year and, hence, should not be associated with our IVs if the independence assumption holds. We did find a small association of year of birth with IV^sep-aug^, which may be due to chance or because, for instance, parents deliberately time the birth of their children and preference for timing has changed over time. However, we adjusted for year of birth, so this association, if real, should not bias the results.

To assess the exclusion restriction assumption, we used forest plots to investigate whether the effect of IVmonths was acting via relative age in school year or seasonality. For example, 3mm asymmetry (right angle) (astigmatism) shows a seasonal pattern rather than a discontinuity at the transition between school years when plotted by month of birth (see Figure S18). That said, date of birth relative to school entry cutoff is a compound treatment as it may affect later outcomes through pathways other than relative age in the classroom. For example, pathways may include years of pre-primary education (or unstructured learning at home) and relative age at the end of compulsory schooling or labour market minimum working age. We could not test the exclusion restriction assumption for pathways other than for seasonality. As a result, our study looks at the intention-to-treat effect of relative age and should be seen as an estimate of the whole compound treatment.

Figure S1: Directed Acyclic Graph of the Assumptions Underpining our Instrumental Variable Analysis


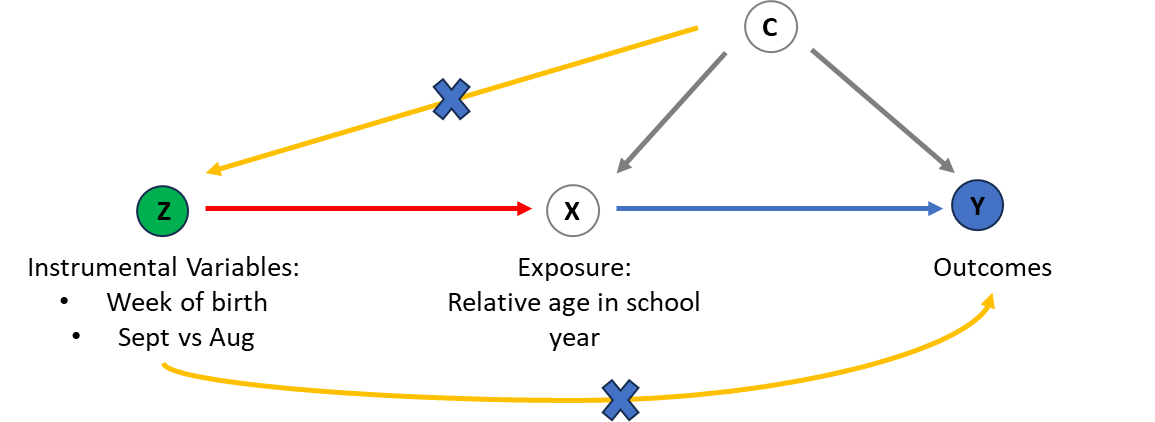


*Note: X and C are unobserved.

Figures S2-S21: Forestplots for all outcomes that passed the Bonferroni threshold for IV^sep-aug^ or IV^weeks^ by month of birth vs September (IV^months^)

Figure S2: Age completed full time education by month of birth vs September

Note: Baseline (0) is month of September. Beta is the association between each month of birth and the outcome, relative to September.

Figure S3: Year ended full time education by month of birth vs September

Note: Baseline (0) is month of September. Beta is the association between each month of birth and the outcome, relative to September.

Figure S4: Have CSEs or equivalent by month of birth vs September

Note: Baseline (0) is month of September. Beta is the association between each month of birth and the outcome, relative to September.

Figure S5: Have O levels or GCSEs or equivalent by month of birth vs September

Note: Baseline (0) is month of September. Beta is the association between each month of birth and the outcome, relative to September.

Figure S6: Have A levels or AS levels or equivalent by month of birth vs September

Note: Baseline (0) is month of September. Beta is the association between each month of birth and the outcome, relative to September.

Figure S7: Have college or university degree by month of birth vs September

Note: Baseline (0) is month of September. Beta is the association between each month of birth and the outcome, relative to September.

Figure S8: Comparative height at age 10 by month of birth vs September

Note: Baseline (0) is month of September. Beta is the association between each month of birth and the outcome, relative to September.

Figure S9: Age started smoking in former smokers by month of birth vs September

Note: Baseline (0) is month of September. Beta is the association between each month of birth and the outcome, relative to September.

Figure S10: Year job started by month of birth vs September

Note: Baseline (0) is month of September. Beta is the association between each month of birth and the outcome, relative to September.

Figure S11: Trouble concentrating on things in last 2 weeks by month of birth vs September

Note: Baseline (0) is month of September. Beta is the association between each month of birth and the outcome, relative to September.

Figure S12: Forced expiratory volume in 1-second (FEV1) Z-score by month of birth vs September

Note: Baseline (0) is month of September. Beta is the association between each month of birth and the outcome, relative to September.

Figure S13: Job code of typist or transcriber by month of birth vs September

Note: Baseline (0) is month of September. Beta is the association between each month of birth and the outcome, relative to September.

Figure S14: Historical job code of typist by month of birth vs September

Note: Baseline (0) is month of September. Beta is the association between each month of birth and the outcome, relative to September.

Figure S15: Sensitivity/Feelings easily hurt by month of birth vs. September

Note: Baseline (0) is month of September. Beta is the association between each month of birth and the outcome, relative to September.

Figure S16: Ever had bowel cancer screening by month of birth vs September

Note: Baseline (0) is month of September. Beta is the association between each month of birth and the outcome, relative to September.

Figure S17: Mood swings by month of birth vs September

Note: Baseline (0) is month of September. Beta is the association between each month of birth and the outcome, relative to September.

Figure S18: 3mm asymmetry angle (right) by month of birth vs September

Note: Baseline (0) is month of September. Beta is the association between each month of birth and the outcome, relative to September.

Figure S19: Mean MO in tapetum on FA skeleton (right) by month of birth vs September

Note: Baseline (0) is month of September. Beta is the association between each month of birth and the outcome, relative to September.

Figure S20: Mean OD in tapetum on FA skeleton (right) by month of birth vs September

Note: Baseline (0) is month of September. Beta is the association between each month of birth and the outcome, relative to September.

Figure S21: Mean MO in inferior cerebellar peduncle on FA skeleton (left) by month of birth vs September

Note: Baseline (0) is month of September. Beta is the association between each month of birth and the outcome, relative to September.

Table S2: Logistic Regression Results to Compare the Covariates of People Born in August vs September.

| **Exposure** | **n** | **OR** | **95% CI** | **p** |
| --- | --- | --- | --- | --- |
| Sex (Male/Female) | 64,075 | 1.0125 | 0.9815, 1.0444 |  |
| Year of birth (Years) | 64,075 | 0.9979 | 0.9960, 0.9998 |  |
| Birth weight (Kg) | 36,652 | 1.0202 | 0.9888, 1.0527 |  |
| UKB Assessment centre (region) | 64,075 |  |  | 0.7457 |
| Intercept |  | 1.0149 | 0.9667, 1.0656 |  |
| 10003 Stockport (pilot) |  | 0.8758 | 0.7366, 1.0408 |  |
| 11001 Manchester |  | 1.0322 | 0.9333, 1.1416 |  |
| 11002 Oxford |  | 0.9631 | 0.8683, 1.0681 |  |
| 11003 Cardiff |  | 0.9457 | 0.8150, 1.0972 |  |
| 11004 Glasgow |  | 0.8694 | 0.6763, 1.1162 |  |
| 11005 Edinburgh |  | 0.9737 | 0.8126, 1.1667 |  |
| 11006 Stoke |  | 0.9768 | 0.8959, 1.0651 |  |
| 11007 Reading |  | 0.9325 | 0.8611, 1.0097 |  |
| 11008 Bury |  | 0.9579 | 0.8865, 1.0349 |  |
| 11009 Newcastle |  | 0.9723 | 0.9052, 1.0445 |  |
| 11011 Bristol |  | 0.9592 | 0.8942, 1.0289 |  |
| 11012 Barts |  | 1.0141 | 0.8984, 1.1448 |  |
| 11013 Nottingham |  | 0.9259 | 0.8601, 0.9966 |  |
| 11014 Sheffield |  | 0.9775 | 0.9060, 1.0546 |  |
| 11016 Liverpool |  | 0.9237 | 0.8578, 0.9946 |  |
| 11017 Middlesborough |  | 0.9805 | 0.9018, 1.0662 |  |
| 11018 Hounslow |  | 0.9872 | 0.9058, 1.0760 |  |
| 11020 Croydon |  | 0.9542 | 0.8766, 1.0387 |  |
| 11021 Birmingham |  | 0.9456 | 0.8706, 1.0270 |  |
| 11022 Swansea |  | 0.8671 | 0.5748, 1.3035 |  |
| 11023 Wrexham |  | 0.5912 | 0.2802, 1.1948 |  |

The outcome sep-aug is a binary variable with August is coded as 0 and September coded as 1. For UKB assessment centre, the baseline is the assessment centre with the largest sample size (Leeds) and the p value is for the likelihood ratio test for a model including assessment centres vs a model without them.
